# Supplementary material for: Discovery and application of insertion-deletion (INDEL) polymorphisms for QTL mapping of early life-history traits in Atlantic salmon
Source: BMC Genomics. 2010 Mar 8;11:156. doi: 10.1186/1471-2164-11-156 (PMC2838853; doi:10.1186/1471-2164-11-156)
Supplement: Additional file 2 — Information on developed 76 locus single-run INDEL panel in Atlantic salmon. Information on fluorescence labeling, primer concentrations, PCR pooling and links to alignments, INDEL motifs and GENESCAN (Burge and Karlin 1997) predictions of genes/exons are available in html format. [file 1471-2164-11-156-S2.ZIP › Additionalfile2/snpsummary3960.html]

```
Cluster 885 Contig 3

prev  Summary    Contig List  next
```

Size of Consensus sequence = 902

Number of sequences = 11

Minimum redundancy = 4

Key

A gi|45308966|gb|CK879335.1|CK879335 SGP143508 Atlantic salmon Gills cDNA library Salmo salar cDNA clone G5-0735 5', mRNA sequence  
B gi|84971392|gb|DW469793.1|DW469793 SGP298672 Atlantic salmon Testis cDNA library Salmo salar cDNA clone MG4-2969 5', mRNA sequence  
C gi|117471896|gb|EG804115.1|EG804115 EST\_ssal\_evd\_1222 ssalevd thymus Salmo salar cDNA Salmo salar cDNA clone ssal\_evd\_006\_042\_fwd 3', mRNA sequence  
D gi|59834113|gb|DN139796.1|DN139796 SGP265803 Atlantic salmon Head kidney cDNA library Salmo salar cDNA clone FN4-3022 5', mRNA sequence  
E gi|89831315|gb|DY693280.1|DY693280 EST\_ssal\_rgb2\_49019 ssalrgb2 mixed\_tissue Salmo salar cDNA Salmo salar cDNA clone ssal\_rgb2\_579\_089\_rev 5', mRNA sequence  
F gi|85037058|gb|DW565236.1|DW565236 EST\_ssal\_rgb2\_29655 rgb2 Salmo salar cDNA clone ssal\_rgb2\_548\_048\_rev 5', mRNA sequence  
G gi|89846087|gb|DY702210.1|DY702210 EST\_ssal\_rgb2\_57949 ssalrgb2 mixed\_tissue Salmo salar cDNA Salmo salar cDNA clone ssal\_rgb2\_593\_195\_rev 5', mRNA sequence  
H gi|89849258|gb|DY705381.1|DY705381 EST\_ssal\_rgb2\_61120 ssalrgb2 mixed\_tissue Salmo salar cDNA Salmo salar cDNA clone ssal\_rgb2\_598\_141\_rev 5', mRNA sequence  
I gi|117490181|gb|EG822398.1|EG822398 EST\_ssal\_evd\_24139 ssalevd thymus Salmo salar cDNA Salmo salar cDNA clone ssal\_evd\_531\_135\_fwd 3', mRNA sequence  
J gi|117454517|gb|EG786736.1|EG786736 EST\_ssal\_evd\_7382 ssalevd thymus Salmo salar cDNA Salmo salar cDNA clone ssal\_evd\_508\_223\_fwd 3', mRNA sequence  
K gi|89871691|gb|DY727814.1|DY727814 EST\_ssal\_rgb2\_83553 ssalrgb2 mixed\_tissue Salmo salar cDNA Salmo salar cDNA clone ssal\_rgb2\_637\_004\_rev 5', mRNA sequence

2 SNPs detected

A B C D E F G H I J K  cosegregation weighted

275 - - . - T T T T - - T   2/2 90.91
276 - - . - T T T T - - T   2/2 90.91
